# Supplementary material for: Is Drinking Alcohol Really Linked to Cardiovascular Health? Evidence from the Kardiovize 2030 Project
Source: Nutrients. 2020 Sep 17;12(9):2848. doi: 10.3390/nu12092848 (PMC7551763; doi:10.3390/nu12092848)

Supplementary material

Is drinking alcohol really linked to cardiovascular health? Evidence from the Kardiovize 2030 Project

Andrea Maugeri[1,2,*], Ota Hlinomaz[1], Antonella Agodi[2], Martina Barchitta[2], Sarka Kunzova[1], Hana Bauerova[1], Ondrej Sochor[1], Jose R. Medina-Inojosa[3], Francisco Lopez-Jimenez[3], Manlio Vinciguerra[1], Gorazd Bernard Stokin[1], Juan Pablo González-Rivas[1,4]

[1] International Clinical Research Center, St Anne's University Hospital, Brno, Czech Republic;

[2] Department of Medical and Surgical Sciences and Advanced Technologies "GF Ingrassia", University of Catania, Catania, Italy.

[3] Division of Preventive Cardiology, Department of Cardiovascular Medicine, Mayo Clinic and Mayo Medical School, Rochester, Minnesota, USA.

[4] Department of Global Health and Population, Harvard T.H. Chan School of Public Health, Boston, USA

[*]Corresponding Author:
Andrea Maugeri, MSc, PhD, Department of Medical and Surgical Sciences and Advanced Technologies "GF Ingrassia", University of Catania, Catania, Italy; International Clinical Research Center, St Anne's University Hospital, Brno, Czech Republic; E-mails: andrea.maugeri@unict.it; andrea.maugeri@fnusa.cz.

Supplementary Table S1. Criteria for Cardiovascular Health Metrics

Supplementary Figure S1. Distribution of cardiovascular health metrics according to drinking wine categories.

Supplementary Figure S2. Distribution of cardiovascular health metrics according to drinking beer categories.

Supplementary Figure S3. Distribution of cardiovascular health metrics according to drinking spirit categories.

Supplementary Table S1. Criteria for Cardiovascular Health Metrics

Metric	Definition
**Smoking status**	**Ideal=** Never or quit > 12 months<br>**Intermediate=** Former ≤12 months<br>**Poor=**Current
**BMI**	**Ideal=** <25 Kg/m2<br>**Intermediate=** 25-29.9 Kg/m2<br>**Poor=** ≥30 Kg/m2
**Physical Activity level**[a]	**Ideal=** ≥150 min/week moderate or ≥75 min/ week vigorous or ≥150 min/ week moderate + vigorous<br>**Intermediate=** 1–149 min/ week moderate or 1–74 min/ week vigorous or 1–149 min/ week moderate + vigorous<br>**Poor=**None
**Diet Score**[b]	**Ideal=** 4-5 components<br>**Intermediate=** 2-3components<br>**Poor=** 0-1 component
**Total Cholesterol**	**Ideal=** <200 mg/dL, without medication or physician diagnosis of Hypercholesterolemia<br>**Intermediate=** 200–239 mg/dL, or treated to <200 mg/dL<br>**Poor=** >/240 mg/dL
**Blood pressure**	**Ideal=** <120/<80 mmHg, without medication or physician diagnosis of Hypertension<br>**Intermediate=** SBP 120–139 or DBP 80–89 mmHg, or treated to <120/<80 mmHg<br>**Poor=** SBP ≥l40 or DBP ≥90mmHg
**Fasting glucose**	**Ideal=** <100 mg/dL, without medication or physician diagnosis of Diabetes<br>**Intermediate=** 100–125 mg/dL, or treated to <100 mg/dL<br>**Poor=** ≥ 126 mg/dL

[a] Physical activity was assessed using the International Physical Activity Questionnaire (Craig CL, Marshall AL, Sjöström M, Bauman AE, Booth ML, Ainsworth BE, Pratt M, Ekelund U, Yngve A, Sallis JF, Oja P. International physical activity questionnaire: 12-country reliability and validity. Med Sci Sports Exerc 2003;35(8):1381-95.)

[b] Food and nutrient intakes were assessed by 24-hours recall; we then categorized achievement of the following components: ≥4.5 cups/d of fruits and vegetables (approximated as ≥4.5 servings/day; ≥3.5 oz servings/week of fish (approximated as ≥two 3–5 oz. servings/week); ≥three 1 oz servings/day of whole grains (approximated as ≥three servings/day); sodium (<1,500 mg/d); and ≤36 oz/week of sugar sweetened beverages (approximated as ≤4 glasses/week)

Abbreviations: BMI, body mass index; SBP, systolic blood pressure; DBP, diastolic blood pressure.

Supplementary Figure S1. Distribution of cardiovascular health metrics according to drinking wine categories.

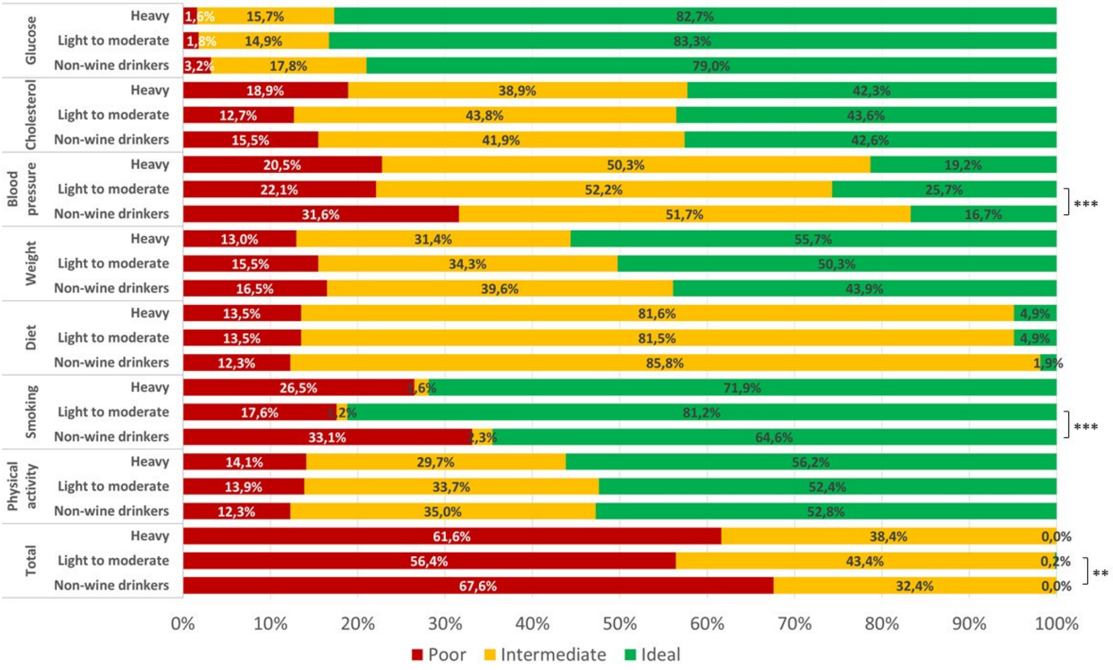

$^{**}p<0.01$, $^{***}p<0.001$ based on the Chi-Squared test using non-wine drinkers as reference group.

Supplementary Figure S2. Distribution of cardiovascular health metrics according to drinking beer categories.

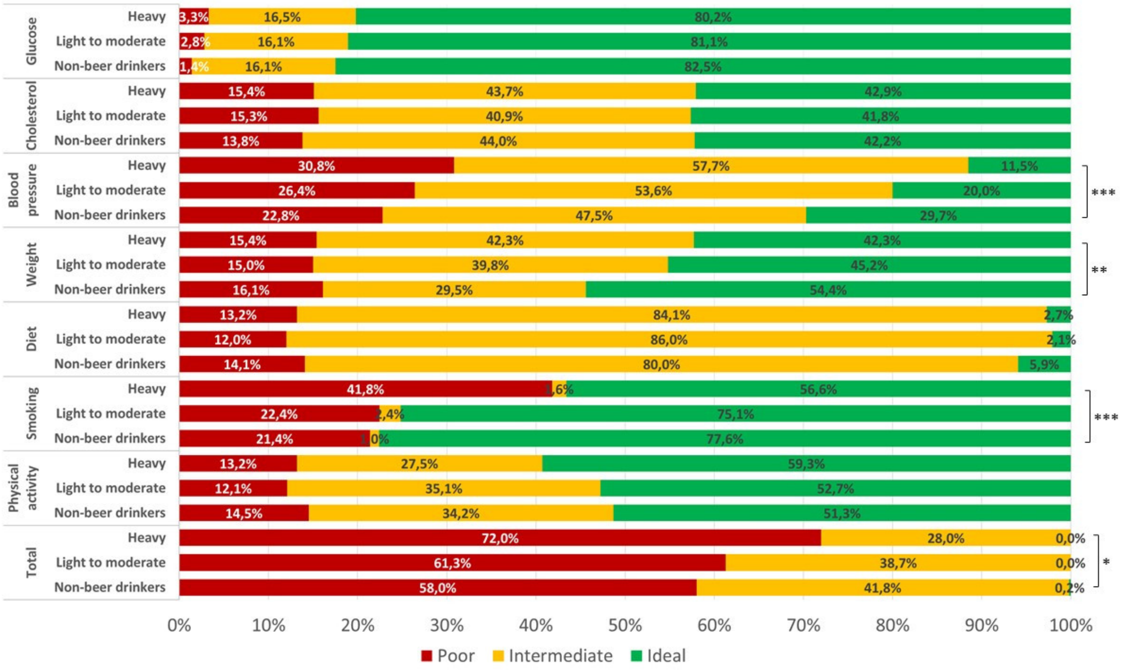

$^{**}p<0.01$, $^{***}p<0.001$ based on the Chi-Squared test using non-beer drinkers as reference group.

Supplementary Figure S3. Distribution of cardiovascular health metrics according to drinking spirit categories.

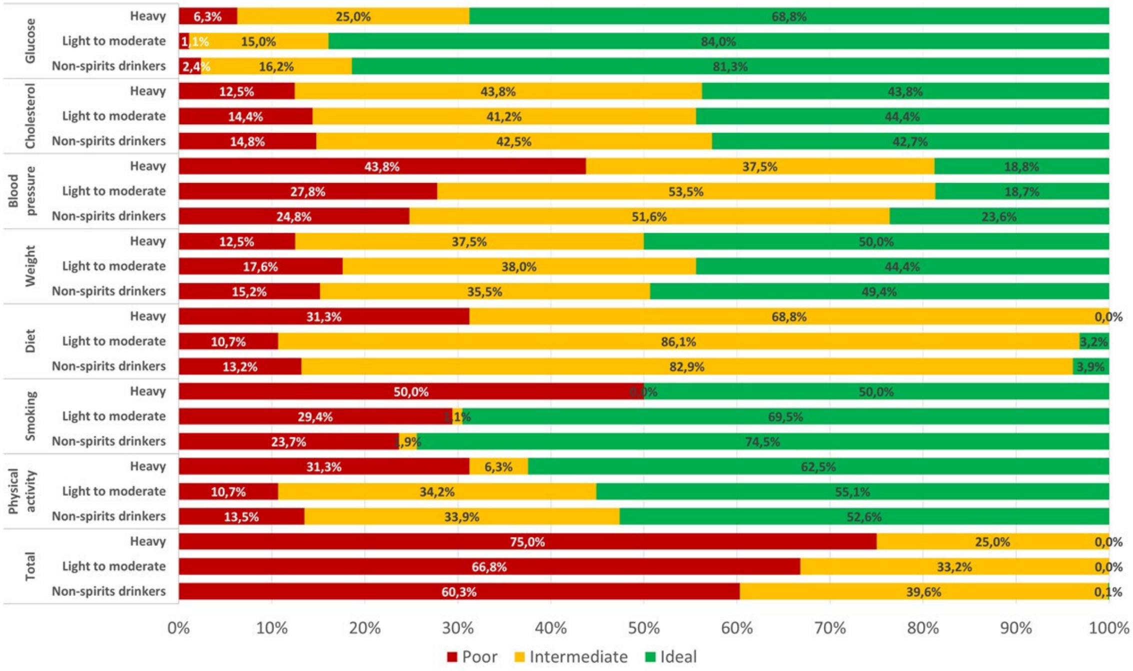

Supplement: Supplementary file 1 [file nutrients-12-02848-s001.pdf]
